# Statistical optimization for simultaneous removal of methyl red and production of fatty acid methyl esters using fresh alga *Scenedesmus obliquus*

**DOI:** 10.1038/s41598-022-11069-z

**Published:** 2022-05-03

**Authors:** Noura El‑Ahmady El‑Naggar, Ragaa A. Hamouda, Ghada W. Abou-El-Souod

**Affiliations:** 1 Department of Bioprocess Development, Genetic Engineering and Biotechnology Research Institute, City of Scientific Research and Technological Applications (SRTA-City), New Borg El‑Arab City, 21934 Alexandria Egypt; 2 Department of Biology, College of Sciences and Arts Khulis, University of Jeddah, Jeddah, Saudi Arabia; 3 Department of Microbial Biotechnology, Genetic Engineering and Biotechnology Research Institute (GEBRI), University of Sadat City, Sadat City, Egypt; 4 Department of Botany and Microbiology, Faculty of Science, Menoufia University, Shibīn al-Kawm, Menoufia Egypt

**Keywords:** Environmental biotechnology, Applied microbiology

## Abstract

Microalgae are a diverse group of microorganisms, the majority of which are photosynthetic in nature. Microalgae have different applications, the most important of which is the biological treatment of wastewater. Microalgae grow in various types of wastewater, such as wastewater polluted by Azo dyes, due to microalgae using wastewater as a culture medium, which contains many nutrients like nitrogen, phosphate, and carbon sources. Microalgae grow in various types of wastewater, such as wastewater polluted by Azo dyes, due to microalgae using wastewater as a culture medium, which contains many nutrients like nitrogen, phosphate, and carbon sources. So, microalgae are used for bioremediation of wastewater due to the efficiency of growing in wastewater and for the high production of lipids followed by trans-esterification to biodiesel. Face-centered central composite design (FCCCD) was used to determine the factors that have the most significant impact on the simultaneous decolorization of methyl red and lipid production by the fresh green alga *Scenedesmus obliquus.* The predicted results indicated that the alga decolorized 70.15% methyl red and produced 20.91% lipids by using 1 g/L nitrogen, an incubation time of 10 days, a pH of 8, and the concentration of methyl red is 17.65 mg/L. The dry biomasses of *S. obliquus* were also examined by SEM and FTIR before and after treatment with methyl red. SEM and FTIR showed that the properties of dry *S. obliquus* were altered after the biosorption of methyl red. According to GC–MS analysis of hexane extracts of *S. obliquus*, the lipid profile differed before and after methyl red decolorization. The results proved that it is possible to use *S. obliquus* to remove dyes and produce renewable fuels such as biodiesel. The novelty of this study is that this is the first time in which the effect of nitrogen concentrations in the medium used for algal growth on the removal of dye has been studied.

## Introduction

The dramatic increase in the global population and hence projects will exceed 9 billion in 2050, which will cause energy shortages and negative effects on the environment due to huge fossil fuel consumption and hence the emission of greenhouse gases^1,2^. Researchers have focused on reducing the requirements of fossil fuels, reducing greenhouse gas emissions, and conserving environmental sustainability^3–6^. Huge quantities of Azo dye are lost during textile industries and other industries, such as pharmaceuticals, cosmetics, food, and paper printing, which lose approximately 10 to 15% of consumed Azo dyes^7,8^. A large amount of wastewater is produced every day because textile industries use several hundred thousand gallons of water every day, which contains many dyes and some heavy metals that cause environmental problems^9,10^. Due to the huge usage of Azo dyes in different industries, Azo dyes are the major compounds of effluents^11^. The occurrence of Azo dyes in aquatic bodies can cause serious disorders and adversely affect human health^12^. Azo dyes cause carcinogenic and mutagenic activities and can cause allergic reactions^13^. Azo dye treatments are a major challenge because they encompass aromatic rings, azonic linkages, and amino groups, which cause multiple damage to the receiving environment^14^. A variety of microorganisms (algae, yeast, fungi, and bacteria) have been investigated for their potential for textile dye bioremediation^15,16^. Algae are used in bioremediation for the following reasons, high growth rates^17,18^, having the ability to sequester CO_2_^17,19^, algal growth is not affected by stress conditions^19^, no competition with food crops^18^, can grow in wastewater and seawater, and can be used in wastewater treatment with both live and dead algae^18,20^.

The efficacy of various algal strains in dye degradation potential has already been shown^21^. Industrial textile wastewater contains necessary nutrients for algae cultivation, including organic dyes as potential sources of carbon, nitrates, and metals as micronutrients^22^. Therefore, textile wastewater seems to be a promising and inexpensive microalgal growing medium^23,24^. Despite their adverse effects on fish, azo dyes in wastewater do not obstruct the progress of certain algal species. However, a few other algal strains in water are very sensitive to azo dyes^25^. In contrast to bacteria, Liu et al.^26^ showed a greater impact of the marine alga *Shewanella* in decolorizing azo dyes even in saline environments. Algal bioremediation seems to have become a promising technology for the treatment of textile wastewater^27^. Since algae are photosynthetic, they obtain energy from sunlight, carbon dioxide, and wastewater nutrients and perform photosynthesis processes^28^. Algae can consume coloured water during their growth, potentially decolorizing dye-contaminated wastewater, and the resulting algal biomass can be processed further for bioenergy and algal-based bioproduct production. Algae fulfil most of the criteria for an applicable dye elimination tool and have the possibility to serve as an encouraging dye removal method in the future^29^.

However, microalgae have recently gained considerable attention because of their capacity for carbon dioxide fixation and bioremediation of textile wastewater^6^. Microalgae can be used simultaneously, first for the bioremediation of textile wastewater and then to accumulate lipids^30^. Some microalgae biomasses contain up to 70% total extractable lipids (g/g dry weight). Biodiesel is derived from lipids produced by microalgae through the transesterification process^31^. Therefore, biodiesel can be used as an environmentally friendly, sustainable fuel in electricity production and can meet the energy needs of the textile sector^32^. Microalgae can use carbon dioxide from the diesel generator atmosphere and organic dyes to photosynthesize it into carbohydrates^33^. Compared with other organic diesel raw materials, microalgae have certain advantages, as their growth rate is rapid, they can be developed approximately everywhere, including sewage, wastewater, saltwater, and arid land, and they do not need fertile agricultural land. Microalgae production is not seasonal and can be harvested daily. Its waste can be used as feed for animals or for other purposes^34^. Various microalgae are considered to have a strong effect on the growth of biomass and the metabolism of fatty acids and lipids^35^. Microalgal oils can be transformed using current technologies into jet fuel, gasoline, and diesel^31^. Many microalgae organisms can be stimulated to produce large amounts of lipids, which leads to high oil production^36^. The most effective stress factor that influences microalgae lipid yield is nitrogen starvation, and the total lipid content of microalgae increases from 25.5 to 45.7% (g/g) under stress conditions^37^. Total and triacylglycerol lipid concentrations were increased when algae biofilms were grown on porous membrane material under nitrogen starvation^38^. The most critical factor that affects microalgal metabolism is pH^39^. In accordance with the pH changes, the production of lipids and biomass changes^40^.

Microalgae, *Chlorella vulgaris* FACHB-8 and *Chlorella* sp. FACHB-31 have been used to remediate anaerobically treated swine wastewater (ADSW), and production biodiesel production, the results showed that both strains were good candidates for biodiesel production and bio-remediate of wastewater^41^.

The objectives of this work were to evaluate the capacity of the live alga *Scenedesmus obliquus* to biodegrade dye (methyl red) and to determine the production of lipids by the algal biomass for future possible applications in biodiesel production and statistical optimization of process variables by face-centered central composite design (FCCCD) for biodegradation of methyl red by *Scenedesmus obliquus*. SEM and FTIR analyses were used to characterize the biomass. GC–MS analysis was also used to determine the profile of the lipids.

## Material and methods

### Material

**Figure 1:**
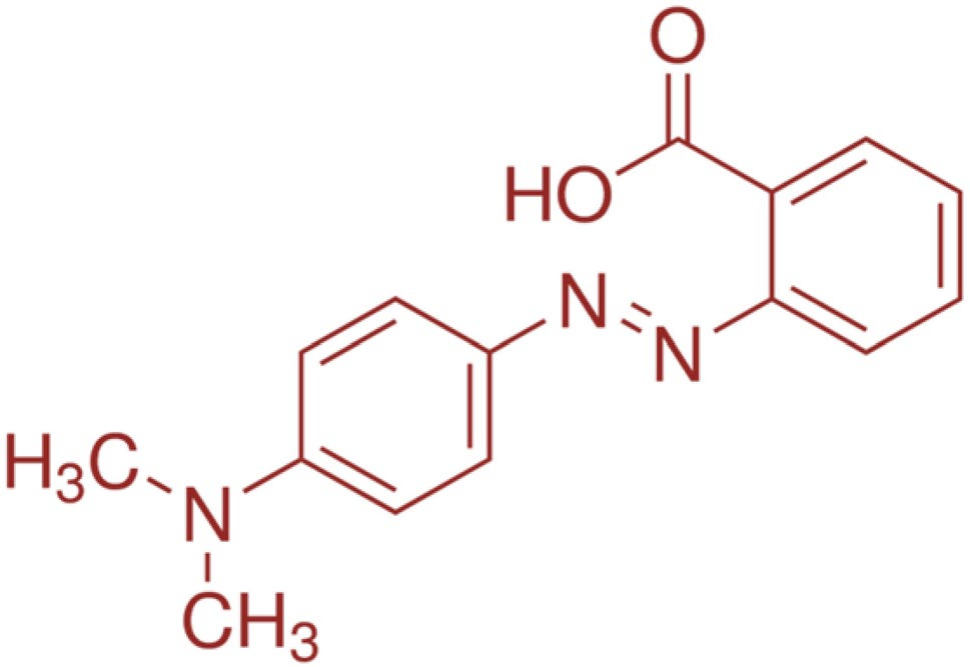
Formula of methyl red (Azo dye).

*Scenedesmus obliquus* microgreen alga was acquired from the “Microbial Biotechnology Department, Genetic Engineering and Biotechnology Research Institute (GEBRI), University of Sadat City, Egypt”. Methyl red was obtained from Sigma–Aldrich, formula as shown in Fig. 1

### Alga culture

*S. obliquus* was grown on a modified Bischoff and bold^42^ basal medium (all components were purchased from sigma Sigma-Aldrich in Egypt)^42^.

### Optimization of process variables by face-centered central composite design (FCCCD)

Ten millilitres of alga at log phase were inoculated in 90 mL of medium using FCCCD to appraise the influence of four factors and investigate their optimum levels on methyl red decolorization and lipid production. Table 1 presents thirty runs with four variables, each of which varies from − 1 (low level), 0 (zero or middle level), and + 1 (high level). The selected independent factors were initial concentration of KNO_3_ as nitrogen source (X_1_: 0.5, 1, 1.5 g/L), incubation time (X_2_: 6, 10, 14 days), initial pH (X_3_: 7, 8, 9), methyl red concentration (X_4_: 10, 20, 30 mg/L) with an intensity of 80 μE m^–2^ s^–1^ continuous light. The relations among the four independent factors in addition to the responses (% methyl red (X_4_) decolorization, biomass and lipid contents were determined using the following second-order polynomial equation:

1 $$Y = \beta_{0} + \sum\limits_{i} {\beta_{i} X_{i} + } \sum\limits_{ii} {\beta_{ii} X_{i}^{2} } + \sum\limits_{ij} {\beta_{ij} X_{i} X_{j} }$$

**Table 1:** Face-centered central composite design matrix of four process variables for methyl red decolorization (%) and lipid production (%) by using *Scenedesmus obliquus*.

Std	Run	Type	X_1_	X_2_	X_3_	X_4_	Methyl red decolorization (%)			Lipid production (%)		
							Actual	Predicted	Residuals	Actual	Predicted	Residuals
1	1	Fact	− 1	− 1	− 1	− 1	29.63	29.31	0.32	18.47	18.44	0.03
7	2	Fact	− 1	1	1	− 1	57.27	56.67	0.60	18.34	18.25	0.09
25	3	Center	0	0	0	0	70.26	70.30	− 0.04	20.80	20.50	0.30
8	4	Fact	1	1	1	− 1	53.80	53.86	− 0.06	20.08	20.04	0.04
20	5	Axial	0	1	0	0	67.57	67.62	− 0.05	19.33	19.41	− 0.08
15	6	Fact	− 1	1	1	1	50.31	50.52	− 0.21	15.00	15.01	− 0.01
14	7	Fact	1	− 1	1	1	56.55	56.87	− 0.32	14.64	14.55	0.09
16	8	Fact	1	1	1	1	41.26	41.71	− 0.44	15.20	15.24	− 0.04
26	9	Center	0	0	0	0	70.50	70.30	0.20	20.57	20.50	0.07
24	10	Axial	0	0	0	1	67.24	66.10	1.14	17.16	17.26	− 0.10
28	11	Center	0	0	0	0	68.50	70.30	− 1.80	20.70	20.50	0.20
10	12	Fact	1	− 1	− 1	1	53.36	54.09	− 0.73	14.66	14.76	− 0.10
23	13	Axial	0	0	0	− 1	63.11	62.81	0.30	21.26	21.29	− 0.03
6	14	Fact	1	− 1	1	− 1	51.53	51.64	− 0.11	19.71	19.74	− 0.03
29	15	Center	0	0	0	0	68.40	70.30	− 1.90	20.34	20.50	− 0.16
27	16	Center	0	0	0	0	70.56	70.30	0.26	20.66	20.50	0.16
18	17	Axial	1	0	0	0	68.30	66.71	1.59	19.36	19.39	− 0.03
4	18	Fact	1	1	− 1	− 1	56.62	57.01	− 0.39	19.70	19.76	− 0.07
13	19	Fact	− 1	− 1	1	1	56.66	56.40	0.26	14.58	14.52	0.06
2	20	Fact	1	− 1	− 1	− 1	41.34	41.36	− 0.02	19.64	19.59	0.05
9	21	Fact	− 1	− 1	− 1	1	47.87	48.04	− 0.17	15.17	15.18	0.00
22	22	Axial	0	0	1	0	67.96	66.97	0.99	20.70	20.87	− 0.17
11	23	Fact	− 1	1	− 1	1	55.58	55.60	− 0.02	15.55	15.53	0.02
19	24	Axial	0	− 1	0	0	64.23	62.74	1.49	19.03	19.08	− 0.05
12	25	Fact	1	1	− 1	1	52.83	52.35	0.48	15.41	15.33	0.08
30	26	Center	0	0	0	0	69.27	70.30	− 1.03	20.33	20.50	− 0.17
17	27	Axial	− 1	0	0	0	64.94	65.09	− 0.15	18.60	18.70	− 0.10
21	28	Axial	0	0	− 1	0	64.81	64.36	0.45	21.10	21.06	0.04
5	29	Fact	− 1	− 1	1	− 1	44.45	45.16	− 0.72	18.12	18.16	− 0.04
3	30	Fact	− 1	1	− 1	− 1	54.33	54.25	0.08	18.36	18.41	− 0.05

	Variable	− 1	0	+ 1								
X_1_	Nitrogen source conc. (g/L)	0.5	1	1.5								
X_2_	Incubation time (days)	6	10	14								
X_3_	Initial pH level	7	8	9								
X_4_	Methyl red concentration (mg/L)	10	20	30								

The symbol Y is the predicted methyl red decolorization (%) or lipid biosorption percentage, the linear coefficient (β_i_), quadratic coefficients (β_ii_), the regression coefficients (β_0_), the coded values of the independent variables (X_i_), and the interaction coefficients (β_ij_).

### Statistical analysis

For experimental design and statistical analysis, the software Design Expert “Version 7 for Windows” was used. STATISTICA Software “Version 8.0, StatSoft Inc., Tulsa, USA” was used to generate three-dimensional surface plots.

### Analytical methods

For each trial of FCCCD, thirty millilitres of algal suspensions were centrifuged, and the filtrates were analysed with a UV–Vis Dual Beam Spectrophotometer UVS-2700 by determining the absorption variations at 430 nm wavelengths to reveal the residual concentrations of MR dye. The efficiency of the fresh alga *S. obliquus* for MR decolorization was verified as a percentage using the following equation:

$${\text{MR removal}} = \left( {{\text{C}}_{{1}} - {\text{C}}_{{2}} /{\text{C}}_{{1}} } \right)*{1}00$$

where C_1_ is the initial concentration of MR and C_2_ is the final concentration after treatments with algal biomass.

### Dry weight estimation

After the limited time (6, 10, and 14 days) was finished, aliquots of 100 ml were centrifuged, and the residue was dried until constant weight in an oven at 60 °C.

### Lipids determination

For each trial of FCCCD, one gram of algal dry weight was mixed with 50 mL of hexane and then stirred with a magnetic stirrer bar for 20 min. The residue was then separated by filtration, and the solvent was evaporated. The extracted lipid was determined as a percentage by using the following equation^43^:

$${\text{Lipid }}\% = \left( {{\text{G}}_{{1}} /{\text{G}}_{{2}} } \right)*{1}00$$

where G_1_ lipids weight, G_2_ dry weight.

### Preparation of fatty acid methyl ester (FAME) content and analysis by GC–MS

20 mg of the lipid extract were weighted and then 0.5 N methanol KOH (2.805 gm KOH in 100 mL methanol) was added, vortexed, and heated at 50 °C for 15 min. The mixture was then cooled and vortexed to mix well. Then, 5 mL of 4 N HCl (3, 4 ml HCl in 100 mL of water) was added and vortexed. Petroleum ether and hexane 1:1, were then added. The upper layer (FAME) was transferred to another tube and dried at 40 °C. 1 mL of hexane was then added to the dried FAME. The FAMEs were then injected and analysed by GC–MS. The sample was injected into GC–MS with a silica capillary column, HP-5MS, and the carrier gas was helium. The GC–MS temperature programme was initiated at 60 °C (2 min) and then increased to 280 °C at an ionizing rate of 8 °C/min. To evaluate the different peaks, Wiley and Wiley Nist mass spectral databases were applied.

### Fourier transform infrared (FTIR) spectroscopy

FTIR spectroscopy is a crucial method for assessing the changes in the active groups present in the algal biomass after and prior to MR decolorization. Dry algal biomass samples were blended with potassium bromide pellets. Then, FTIR spectra were analysed at 400–4000 cm^−1^ using a Thermo Fisher Nicolete IS10 spectrophotometer (USA).

### Scanning electron microscopy (SEM)

To study the cell surface of *S. obliquus*, the dry biomass was examined with SEM after and prior to MR decolorization. Using an accelerated beam voltage of 30 keV, the gold-coated dry biomass was examined at different magnifications.

## Results and discussion

In this study, the impact of four variables, namely, nitrogen source conc. (g/L), incubation time (days), initial pH level, and methyl red concentration (mg/L) on simultaneous methyl red decolorization and lipid production (as a response) using the microalga *S. obliquus* was investigated.

### Statistical optimization of lipid production and MR removal by the fresh alga, *S. obliquus*

The fermentation medium contents and various environmental conditions (i.e., nutrients, pH, etc.) required for microbial growth play an incredible role in achieving maximum metabolite production^44,45^. The conventional strategy for culture medium optimization (single variable optimization method) has been expanded for optimization of the process, but this method not only takes a long time, is expensive and laborious, but also ignores the effects of independent factor interactions^46,47^.

The statistical designs of experiments have been used for the optimization of process parameters and can be performed in two key steps: first, screening of the significant variables and second, optimization of those variables. These designs have several advantages, including fewer experimental runs for multiple parameters; avoiding misinterpretation of the results that occurs with the conventional strategy; assisting in determining the optimum level of each variable; exploring the individual, quadratic, and interaction effects between different variables; and predicting the optimum conditions for maximum response^48^. To determine the effects of four process variables and to obtain their optimum values for maximum simultaneous lipid production and decolorization of MR using fresh alga, *S. obliquus*, an overall number of thirty experimental runs of FCCCD were done. Table 1 shows the observed and predicted results for both lipid production and decolorization of MR. The results indicate substantial variations in both lipid production and MR decolorization percentages by *S. obliquus* according to the combinations of the four variables. Based on the data collected, the percentage of methyl red decolorization varied significantly from 29.63 to 70.56%.

These results agree with those of Abou-El-Souod et al.^49^, in which the decolorization percentage of methyl red (20 ppm) by *S. obliquus* was 48.60%. The decolorization percentage of methyl red (20 ppm) by *C. vulgaris* was in the range of 71%^21^. The result demonstrated that lipid production by *S. obliquus* ranged from 14.52 to 21.29% of dry weight, as shown in Table 1. The lipid production percentage of dry weight *S. obliquus* ranged from 11 to 55%^19^, 15.2 to 24.4%^50^, 21 to 42%^35^, 20.4 to 25.2%^51^. The lipid yield percentage of dry weight *Scenedesmus dimorphus* was 26^35^, *Scenedesmus rubescens* ranged from 18.5 to 23.2%^52^, *Scenedesmus quadricauda* was 18.4^53^. The highest levels of methyl red decolorization (70.56%) were obtained in run no. 16 when all the tested variables were at their central value: the nitrogen source concentration was set at 1 g/L, the incubation time (10 days), initial pH level (8), and methyl red concentration (20 mg/L). Meanwhile, the highest levels of lipid production (21.29%) were shown in run no. 13, when the nitrogen source concentration was set at 1 g/L, the incubation time (10 days), initial pH level (10), and methyl red concentration (10 mg/L). The minimum methyl red decolorization (29.63%) was obtained in run no. 1 when the nitrogen source concentration was set at 0.5 g/L, the incubation time (6 days), initial pH level (7) and the methyl red concentration was 10 mg/L. The minimum lipid production was 14.58% when the nitrogen source concentration was set at 0.5 g/L, the incubation time (6 days), initial pH level (9) and methyl red concentration (30 mg/L).

### Multiple regression analysis and ANOVA for methyl red decolorization by fresh *S. obliquus*

The FCCCD results for methyl red decolorization by fresh *S. obliquus* were analysed statistically, employing multiple regression analysis and analysis of variance (ANOVA), and the results of the analyses are presented in Tables 2, 3. Statistical regression analysis data such as adj R^2^ value, coefficient (R^2^) value, predicted R^2^ value, the main influence of each variable, lack of fit, *F*-value, and probability *P-*value were investigated to determine the model reliability. Individual, interactions and quadratic effects of the different variables were also evaluated.

**Table 2:** ANOVA for FCCCD results for methyl red decolorization (%) by using *Scenedesmus obliquus*.

Source of variance		Sum of squares	*df*	Mean square	*F* value	*P-*value *P*rob > *F*	Coefficient estimate
Model		3172.48	14	226.61	191.87	< 0.0001*	70.30
Linear effects	X_1_—(nitrogen source conc.)	11.75	1	11.75	9.95	0.0065*	0.81
	X_2_—(incubation time)	107.34	1	107.34	90.88	< 0.0001*	2.44
	X_3_—(initial pH level)	30.51	1	30.51	25.84	0.0001*	1.30
	X_4_—(methyl red concentration)	48.64	1	48.64	41.18	< 0.0001*	1.64
Interaction effects	X_1_ X_2_	86.29	1	86.29	73.06	< 0.0001*	− 2.32
	X_1_ X_3_	31.02	1	31.02	26.27	0.0001*	− 1.39
	X_1_ X_4_	36.09	1	36.09	30.55	< 0.0001*	− 1.50
	X_2_ X_3_	180.30	1	180.30	152.66	< 0.0001*	− 3.36
	X_2_ X_4_	302.08	1	302.08	255.77	< 0.0001*	− 4.35
	X_3_ X_4_	56.19	1	56.19	47.58	< 0.0001*	− 1.87
Quadratic effects	X_1_^2^	50.18	1	50.18	42.48	< 0.0001*	− 4.40
	X_2_^2^	67.89	1	67.89	57.48	< 0.0001*	− 5.12
	X_3_^2^	55.68	1	55.68	47.14	< 0.0001*	− 4.64
	X_4_^2^	88.49	1	88.49	74.92	< 0.0001*	− 5.84
Error effects	Lack of fit	12.81	10	1.28	1.31	0.4053	
	Pure error	4.91	5	0.98			
Std. dev.		1.09	R-squared			0.9944	
Mean		58.30	Adj R-squared			0.9893	
C.V. %		1.86	Pred R-squared			0.9819	
PRESS		57.76	Adeq precision			53.34

**Table 3:** ANOVA for FCCCD results for lipid production (%) by using *Scenedesmus obliquus*.

Source of variance		Sum of squares	*df*	Mean square	*F* value	*P* value *P*rob > *F*	Coefficient estimate
Model		152.41	14	10.89	496.82	< 0.0001	20.50
Linear effects	X_1_—(nitrogen source conc.)	2.13	1	2.13	97.37	< 0.0001	0.34
	X_2_—(incubation time)	0.49	1	0.49	22.19	0.0003	0.16
	X_3_—(initial pH level)	0.16	1	0.16	7.28	0.0165	− 0.09
	X_4_—(methyl red concentration)	73.25	1	73.25	3342.89	< 0.0001	− 2.02
Interaction effects	X_1_ X_2_	0.04	1	0.04	2.00	0.1777	0.05
	X_1_ X_3_	0.19	1	0.19	8.70	0.0100	0.11
	X_1_ X_4_	2.43	1	2.43	110.95	< 0.0001	− 0.39
	X_2_ X_3_	0.02	1	0.02	0.69	0.4192	0.03
	X_2_ X_4_	0.15	1	0.15	7.00	0.0183	0.10
	X_3_ X_4_	0.13	1	0.13	6.09	0.0261	− 0.09
Quadratic effects	X_1_^2^	5.48	1	5.48	250.16	< 0.0001	− 1.45
	X_2_^2^	4.07	1	4.07	185.73	< 0.0001	− 1.25
	X_3_^2^	0.56	1	0.56	25.38	0.0001	0.46
	X_4_^2^	3.89	1	3.89	177.53	< 0.0001	− 1.23
Error effects	Lack of fit	0.14	10	0.01	0.37	0.9157	
	Pure error	0.19	5	0.04			
Std. dev.		0.15	R-squared			0.9978	
Mean		18.42	Adj R-squared			0.9958	
C.V. %		0.80	Pred R-squared			0.9938	
PRESS		0.94	Adeq precision			64.67	

The model determination coefficient (R^2^) for methyl red decolorization by fresh *S. obliquus* was 0.9944, meaning that 99.44% of the variance in methyl red decolorization was explained by the independent aspects used, and the model could not elucidate just 0.56% of the overall variance. A regression model with a high R^2^ value above 0.9 is considered to have the strongest significant correlation^54,55^. In addition, the Adj R^2^ value of the methyl red decolorization % by fresh *S. obliquus* (Adj R^2^ 0.9893) was very high, which verified that the model is very significant, as shown in Table 2. On the other hand, there is reasonable agreement between the predicted R^2^ value of 0.9819 and the Adj R^2^ value of 0.9893, meaning a strong agreement between the predicted and experimental values of methyl red decolorization percentages. The model used in this study is therefore appropriate to predict methyl red decolorization by fresh *S. obliquus* in the range of independent parameters.

A considerably small value of the coefficient of variation % (C.V. = 1.86%) displays the high accuracy and consistency of the experimental values of methyl red decolorization percentages^56^. Adequate precision determines the level of noise; a level higher than 4 is superior and implies model consistency. The current adequate precision ratio for methyl red decolorization by the fresh *S. obliquus* model is 53.34, which implies model reliability. The standard deviation (SD) of methyl red decolorization by the fresh *S. obliquus* model was 1.09. The PRESS (expected residual sum of squares) value was 57.76, and the mean value of the methyl red decolorization model was 58.30 (Table 2).

The ANOVA of the regression model of methyl red decolorization indicates that the model terms are highly significant, as is apparent from the *F* (Fisher’s variance) value (*F* value = 483.09) and a very minor probability value [*P-*value˂ 0.0001] (Table 3). *P-*values were manipulated as a tool to evaluate the significance of each variable. In this study, the variables with *P-*values of less than 0.05 were found to have significant effects^57,58^. Meanwhile, the lack of fit for methyl red decolorization % was not significant (*P-*value = 0.4053 and *F-*value = 1.31) (Table 3).

The negative coefficient values of the linear, mutual interactions and quadratic effects of the selected process variables denote an antagonistic correlation among the selected process parameters and methyl red decolorization by fresh *S. obliquus* (the variables exert a negative effect). However, the positive coefficient values indicate a synergistic correlation between the selected process parameters and methyl red decolorization by fresh *S. obliquus*. It can be investigated from the values of coefficients that the linear coefficients of X_1_, X_2_, X_3,_ and X_4_ are highly significant. The *P-*values of the coefficients indicate that the relations between X_1,_ and X_2_; X_1_X_3_; X_1_X_4_; X_2_X_3_; X_2_X_4_; X_3_X_4_; X_1_^2^, X_2_^2^, X_3_^2^_,_ and X_4_^2^ had a very significant impact on methyl red decolorization (*P*-value < 0.05).

The polynomial regression equation expressed in terms of coded levels of the variables reveals the mathematical relationships between the independent variables and can be used to make predictions of methyl red decolorization by *S. obliquus* (Y) for given levels of each variable. The mathematical relationship is assumed by the following polynomial equation of the second order:

$$\begin{aligned} {\text{Y}} & = {7}0.{3}0 + 0.{\text{81X}}_{{1}} + {2}.{\text{44X}}_{{2}} + {1}.{3}0{\text{X}}_{{3}} + {1}.{\text{64X}}_{{4}} \hfill \\ & \quad - { 2}.{\text{32X}}_{{1}} {\text{X}}_{{2}} - {1}.{\text{39X}}_{{1}} {\text{X}}_{{3}} - {1}.{5}0{\text{X}}_{{1}} {\text{X}}_{{4}} - { 3}.{\text{36X}}_{{2}} {\text{X}}_{{3}} - {4}.{\text{35X}}_{{2}} {\text{X}}_{{4}} \hfill \\ & \quad- {1}.{\text{87X}}_{{3}} {\text{X}}_{{4}} - { 4}.{4}0{\text{X}}_{1}{^{2}} - {5}.{\text{12X}}_{2}{^{2}} - { 4}.{\text{64X}}_{3}{^{2}} - { 5}.{\text{84X}}_{4}{^{2}} \hfill \\ \end{aligned}$$

where Y is the predicted value of methyl red decolorization % by vital *S. obliquus*. X_1_ is the coded value of the nitrogen source conc., (g/L), X_2_ is the coded value of incubation time (days), X_3_ is the coded value of initial pH level, and X_4_ is the coded value of methyl red concentration (mg/L).

The summary fit results in Table 3 demonstrate that the quadratic polynomial model has the most significant model terms with a very low *P-*value < 0.0001 and a nonsignificant lack of fit with a high probability value (*P-*value = 0.4053) and *F-*value = 1.09.

### Multiple regression analysis and ANOVA for lipid production by fresh *S. obliquus*

Multiple regression analysis and analysis of variance (ANOVA) were used to analyse the FCCCD results for lipid production by fresh *S. obliquus*. The results are shown in Tables 4, 5. The model determination coefficient (R^2^) for lipid production by fresh *S. obliquus* was 0.9978, meaning that 99.78% of the variance in lipid production was explained by the independent factors used, and the model could not elucidate just 0.22% of the overall variance. In addition, the Adj R^2^ value of lipid production by fresh *S. obliquus* (Adj R^2^ 0.9958) was very sharp, which verified great model significance, as shown in Table 4. On the other hand, there was reasonable agreement between the predicted R^2^ value of 0.9938 and the Adj R^2^ value of 0.9958, indicating great harmony between the predicted and experimental values of lipid production by the fresh alga *S. obliquus*. The model used in this study is therefore appropriate to predict lipid production by fresh *S. obliquus* in the range of independent parameters.

**Table 4:** Fit summary of the experimental results of CCD for methyl red decolorization (%) by using *Scenedesmus obliquus*.

Sequential model sum of squares					
Source	*SS*	*Df*	*MS*	*F* value	*P* value
Linear vs mean	198.25	4	49.56	0.41	0.7968
2FI vs linear	691.97	6	115.33	0.95	0.4820
Quadratic vs 2FI	2282.27	4	570.57	483.09	< 0.0001*

Lack of fit tests					
Source	*SS*	*Df*	*MS*	*F* value	*P* value
Linear	2987.05	20	149.35	152.20	< 0.0001*
2FI	2295.08	14	163.93	167.06	< 0.0001*
Quadratic	12.81	10	1.28	1.31	0.4053

Model summary statistics					
Source	SD	R^2^	Adjusted R^2^	Predicted R^2^	PRESS
Linear	10.94	0.0621	− 0.0879	− 0.4248	4545.25
2FI	11.00	0.2790	− 0.1004	− 1.8497	9091.05
Quadratic	1.09	0.9944	0.9893	0.9819	57.76

*Significant values, *Df*: degree of freedom, PRESS: sum of squares of prediction error, 2FI: two factors interaction, SD: Standard deviation, *SS*: Sum of Squares, *MS*: Mean Square.

**Table 5:** Fit summary for experimental results of CCD for lipid production (%) by using *Scenedesmus obliquus*.

Sequential model sum of squares					
Source	*SS*	*Df*	*MS*	*F* value	*P* value
Linear vs mean	76.03	4	19.01	6.19	0.0013*
2FI vs linear	2.97	6	0.49	0.13	0.9914
Quadratic vs 2FI	73.41	4	18.35	837.57	< 0.0001*

Lack of fit tests					
Source	*SS*	*Df*	*MS*	*F* value	*P* value
Linear	76.52	20	3.83	101.14	< 0.0001*
2FI	73.55	14	5.25	138.88	< 0.0001*
Quadratic	0.14	10	0.01	0.37	0.9157

Model summary statistics					
Source	SD	R^2^	Adjusted R^2^	Predicted R^2^	PRESS
Linear	1.75	0.4978	0.4174	0.2881	108.73
2FI	1.97	0.5172	0.2631	− 0.7798	271.83
Quadratic	0.15	0.9978	0.9958	0.9938	0.94

*Significant values, *Df*: degree of freedom, PRESS: sum of squares of prediction error, 2FI: two factors interaction, SD: standard deviation, *SS*: sum of squares, *MS*: mean square.

A considerably small value of the coefficient of variation % (C.V. = 0.80%) displays the high accuracy and consistency of the experimental values of lipid production. The current adequate precision ratio for lipid production by the fresh *S. obliquus* model is 64.67, which implies the high precision and reliability of the model. The Std. Dev. of lipid production by the fresh *S. obliquus* model was 0.15. The PRESS value was 0.94, and the mean value of the lipid production model was 18.42 (Table 4).

The ANOVA of the regression model of lipid production indicates that the model terms are highly significant, as is apparent from the *F* (Fisher’s variance) value (*F-*value = 483.09) and a very minor probability value [*P-*value˂ 0.0001] (Table 5). Meanwhile, the lack of fit for lipid production was not significant (*P-*value = 0.9157 and *F-*value = 0.37) (Table 5).

It can be seen from the coefficients that the linear coefficients of X_1_, X_2_, X_3,_ and X_4_ are highly significant. The *P-*values of the coefficients indicate that the relationships between X_1_ and X_3_; X_1_X_4_; X_2_X_4_; X_3_X_4_; X_1_^2^, X_2_^2^, X_3_^2^_,_ and X_4_^2^ had a very significant impact on lipid production (*P-*value < 0.05). The *P-*value of coefficients indicates that the relations between X_1_ and X_2_ and X_2_X_3_ had a nonsignificant impact on lipid production (*P-*value ˃ 0.05).

Table 5 shows the fit summary results, which indicate that the quadratic polynomial model has the most significant model terms with a very low *P-*value < 0.0013 and a nonsignificant lack of fit with an elevated probability value (*P-*value = 0.9157) and an *F-*value = 0.37. The quadratic polynomial model has the highest R^2^(0.9978), adjusted R^2^(0.9958), and predicted R^2^ (0.9938).

The polynomial regression equation in terms of coded levels of the variables indicates the mathematical relations between the independent variables and can be used to make predictions of lipid production by *S. obliquus* (Y) for given levels of each variable. The mathematical relationship is assumed by the following polynomial equation of the second order:

$$\begin{aligned} {\text{Y}} & = 20.50 \, + \, 0.34{\text{ X}}_{1} + 0.16{\text{ X}}_{2} - 0.09{\text{ X}}_{3} - \, 2.02{\text{ X}}_{4} \hfill \\ & \quad + 0.05{\text{ X}}_{1} {\text{X}}_{2} + 0.11{\text{ X}}_{1} {\text{X}}_{3} - 0.39{\text{ X}}_{1} {\text{X}}_{4} + \, 0.03{\text{ X}}_{2} {\text{X}}_{3} \hfill \\ & \quad + \, 0.10{\text{ X}}_{2} {\text{X}}_{4} - 0.09{\text{ X}}_{3} {\text{X}}_{4} - 1.45{\text{ X}}_{1}{^{2}} - 1.25{\text{ X}}_{2}{^{2}} + 0.46{\text{ X}}_{3}{^{2}} - \, 1.23{\text{X}}_{4}{^{2}} \hfill \\ \end{aligned}$$

where Y is the predicted value of lipid production by vital *S. obliquus*. X_1_: is the coded value of the nitrogen source conc. (g/L), X_2_ is the coded value of the incubation time (days), X_3_ is the coded value of the initial pH level, and X_4_ is the coded value of the methyl red concentration (mg/L).

### The adequacy of the model fit for methyl red decolorization (%) by using *S. obliquus* results

**Figure 2:**
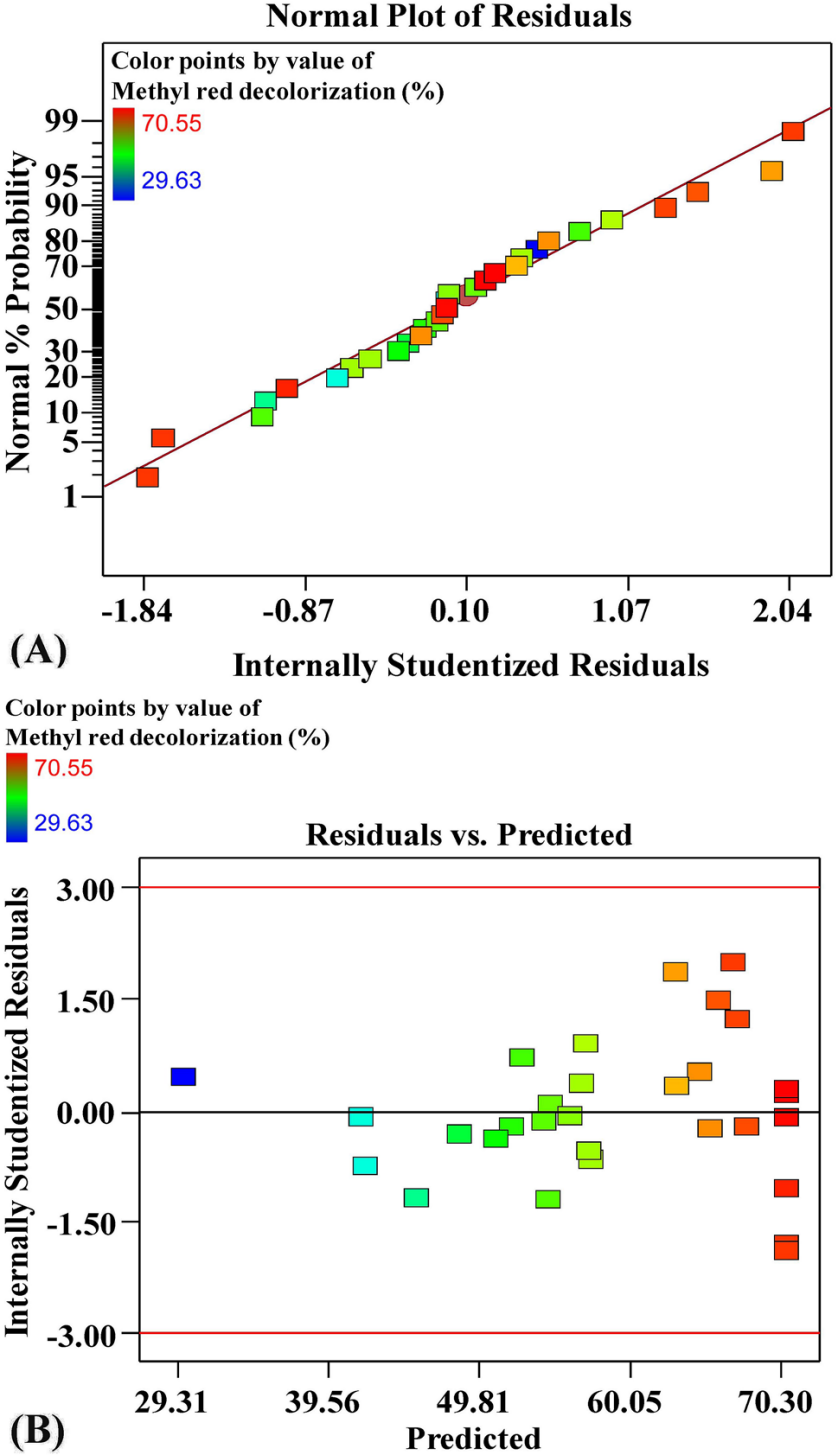
(**A**) Normal probability plot of internally studentized residuals, (**B**) plot of internally studentized residuals versus predicted values of methyl red decolorization (%) by using *Scenedesmus obliquus* as determined by a second-order polynomial equation.

The normal probability plot of internally studentized residuals (NPP) is an important statistical tool for detecting the appropriateness of the model and clarifying whether a set of data is normal or deviates from normality^59,60^. Figure 2A shows the NPP of internally studentized residuals for methyl red decolorization (%) by using *S. obliquus* results in data analysis, which indicates that the residuals are normally disseminated; they are located along the straight diagonal line of the normal distribution of methyl red decolorization (%) by using *S. obliquus,* which reveals suitability of the model, and the predicted results of methyl red decolorization (%) by using *S. obliquus* were well fitted with the experimental results. Figure 2B shows the predicted values of methyl red decolorization (%) by using *S. obliquus* versus the internally studentized residuals as determined by a second-order polynomial equation. Figure 2B shows that the residual data is distributed equally above and below the x-axis, confirming the validity of the model.

### The adequacy of the model fit for lipid production (%) by using *S. obliquus*

**Figure 3:**
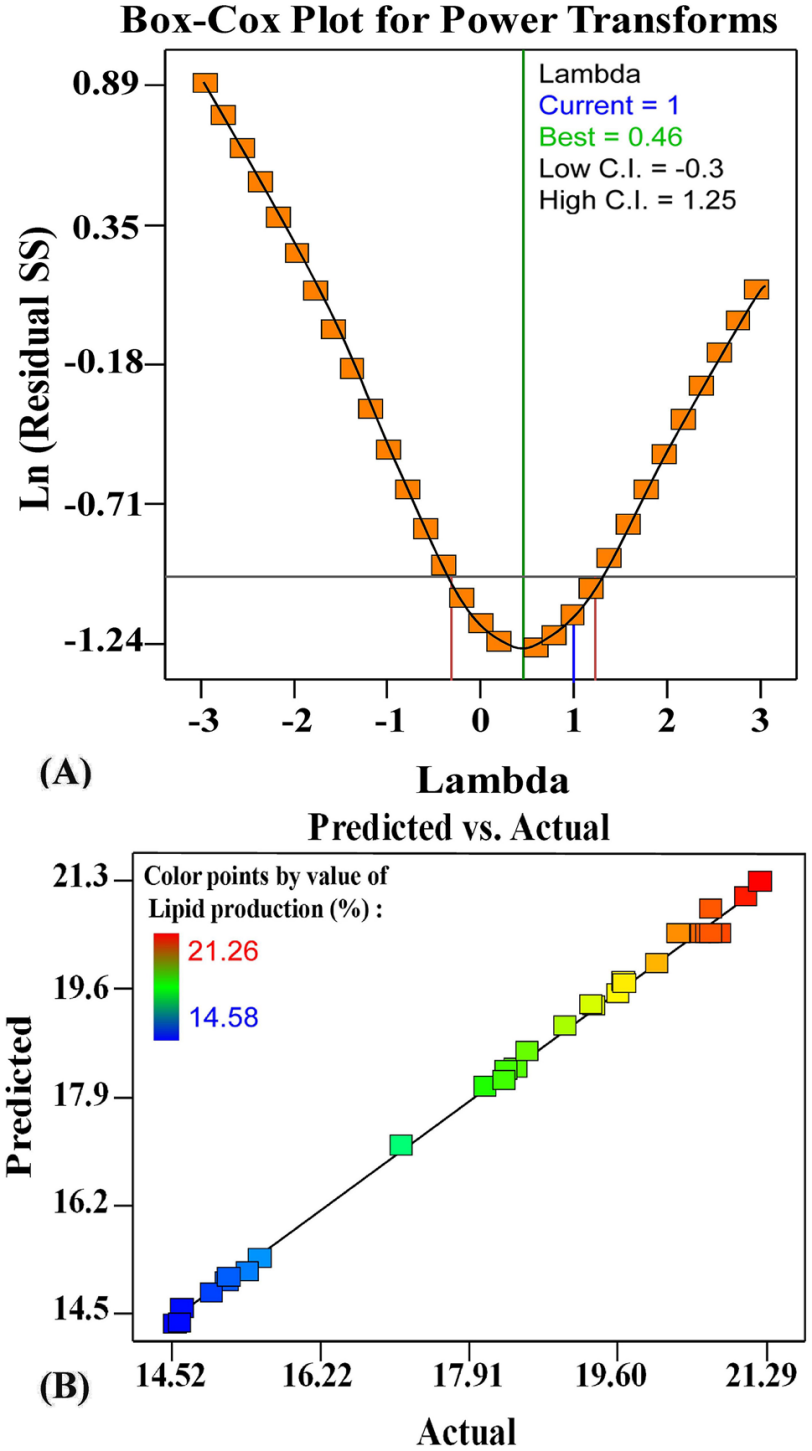
(**A**) Box–Cox plot of model transformation and (**B**) plot of predicted versus actual values of lipid production (%) by using *Scenedesmus obliquus* as determined by a second-order polynomial equation.

Figure 3A displays the graph of the box-Cox plot of the model alteration of lipid production (%) by using *S. obliquus* determined by a second-order polynomial equation. As shown in Fig. 3A, the best value of lambda (λ = 0.46) is located between the two vertical red lines so that no data alteration is necessitated. The red lines indicate the minimum and maximum 95% confidence interval values. Figure 3B presents a plot of predicted versus experimental (actual) values of lipid production (%) by using *S. obliquus*. The graph shows the points adjacent to the diagonal line, revealing a good correlation between the predicted and actual experimental values.

### Three-dimensional surface plots to imagine the effects of independent process factors on methyl red decolorization by using *S. obliquus*

**Figure 4:**
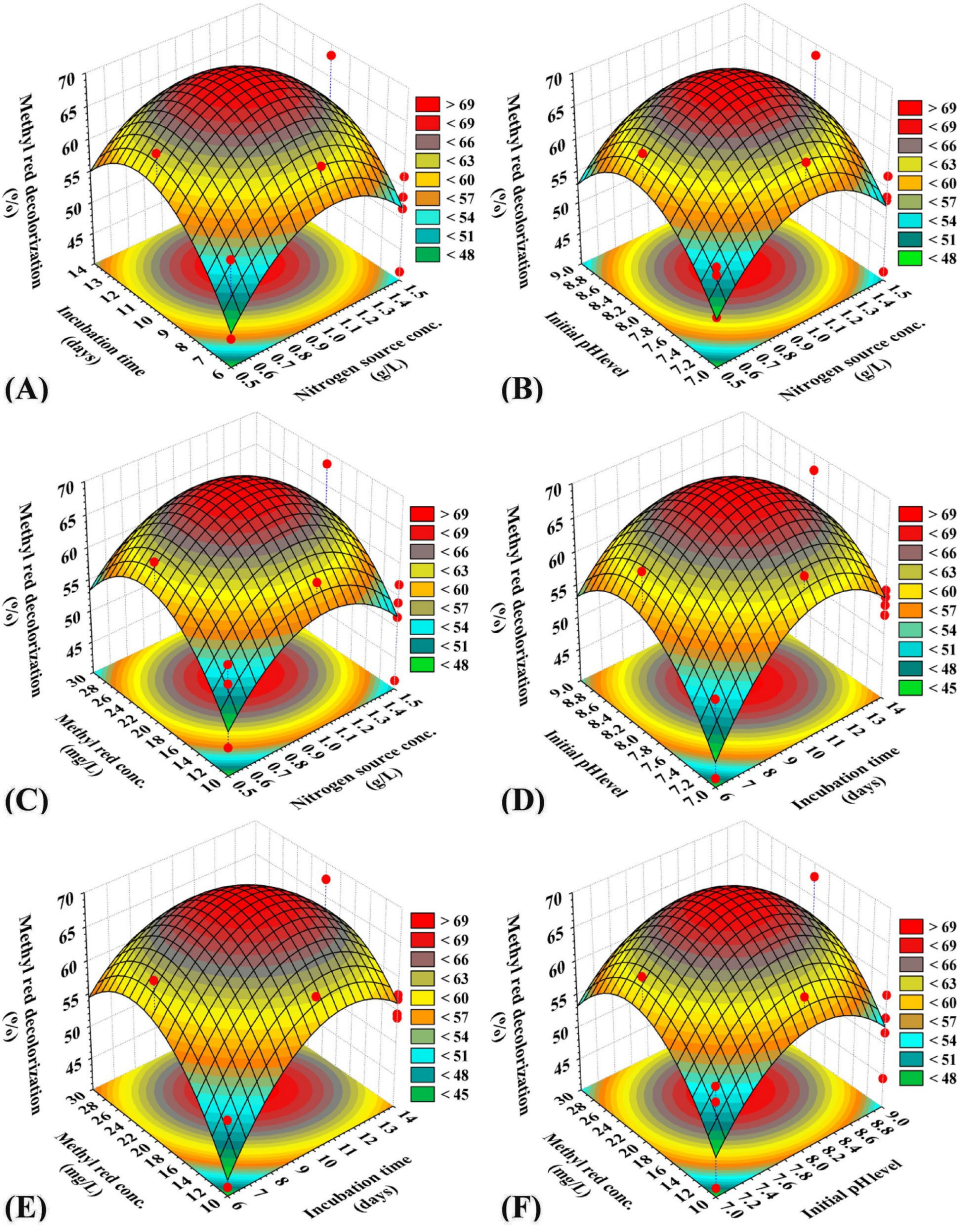
Three-dimensional surface plot for methyl red decolorization (%) by using *Scenedesmus obliquus*, showing the interactive effects of the tested variables.

The three-dimensional (3D) surface plots were generated to recognize the optimum conditions for the highest percentage of methyl red decolorization by using *S. obliquus* and to visualize the effects of the interactions between the selected process factors on the percentage of methyl red decolorization. 3D graphs for the four variables combined in pairs (nitrogen sources conc. (X_1_), incubation time (X_2_), initial pH level (X_3_) and methyl red concentration (X_4_) were created by plotting the percentage of methyl red decolorization on the Z-axis versus two process factors, while other independent process factors were fixed at their center levels. The 3D graph (Fig. 4) illustrates the effect of the nitrogen source conc. (X_1_) and incubation time (X_2_) on methyl red decolorization on the Z-axis, whereas the initial pH level (X_3_) and methyl red concentration (X_4_) were maintained at their center levels.

Figure 4A shows that methyl red decolorization increases with increasing nitrogen source conc., and incubation time. The highest percentage of methyl red decolorization was apparently placed close to the central level of the nitrogen source concentration and incubation time. The center level nitrogen (X_1_) causes a high degradation value of methyl red by S*. obliquus.* Lower and higher concentrations of nitrogen (X_1_) and incubation time (X_2_) resulted in lower methyl red decolorization percentages. By solving the Eq. (1) and analysing Fig. 4A, the maximum methyl red decolorization predicted value of 70.56% could be achieved at the optimal predicted levels of nitrogen source conc. of 1 g/L, and incubation time of 10 days by using *S. obliquus* at initial pH level 8, and 20 mg/L methyl red. Figure 4B represents the influence of nitrogen concentration (X_1_) and initial pH (X_3_) levels on the methyl red decolorization percentages, where the incubation time (X_2_) and methyl red concentrations (X_4_) were maintained at their centre levels. Figure 4C denotes the influence of nitrogen concentration (X_1_) and methyl red concentration (X_4_) on the methyl red decolorization percentages. When the other two variables were at the centre levels, the high and low levels of the tested variables did not affect the methyl red decolorization percentages when using fresh *S. obliquus.* The influence of incubation time (X_2_) and initial pH (X_3_) on the methyl red decolorization percentages is shown in Fig. 4D. As shown in Fig. 4D, the center value of incubation time (X_2_) and initial pH (X_3_) caused the elevation of methyl red decolorization, while the other two variable factors were also at the center value. Figure 4E shows the influence of both incubation time (X_2_) and methyl red concentration (X_4_) on methyl red decolorization percentages by using fresh *S. obliquus*. The highest decolorization was obtained when the nitrogen concentration (X_1_) and initial pH (X_3_) were at their center values. The effect of initial pH (X_3_) and methyl red concentrations (X_3_) on the methyl red decolorization percentages by using fresh *S. obliquus* is shown in Fig. 4F. The low and high values of the initial pH and methyl red concentrations did not increase the percentage of methyl red decolorization when nitrogen concentration and incubation time were maintained at their center levels.

### Three-dimensional surface plots to envision the effects of independent process factors on lipid production % by using *S. obliquus*

**Figure 5:**
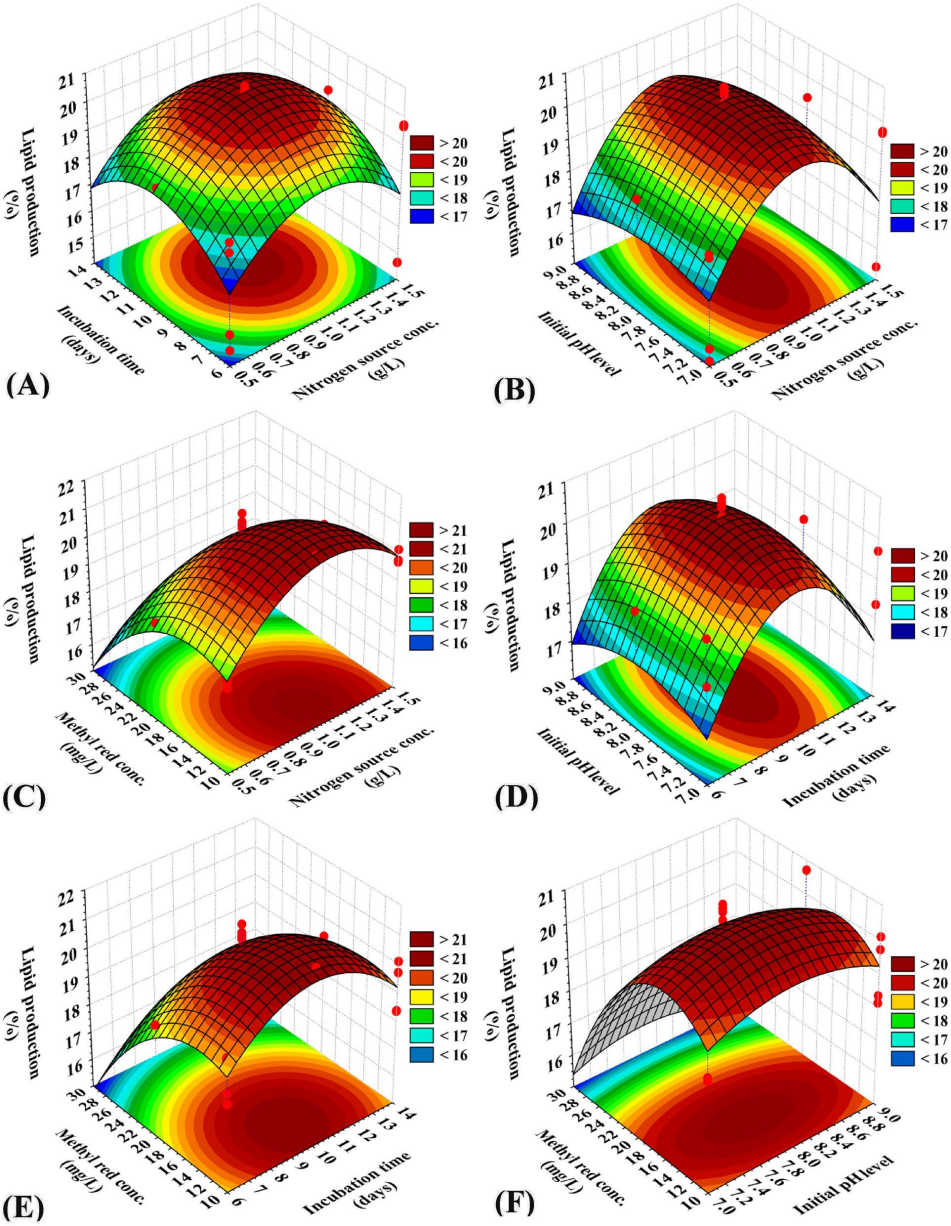
Three-dimensional surface plot for lipid production (%) by using *Scenedesmus obliquus*, showing the interactive effects of the tested variables.

Figure 5 demonstrates the three-dimensional plots for lipid production percentages as a function of nitrogen source conc. (X_1_), incubation time (X_2_), initial pH level (X_3_), and methyl red concentration (X_4_). Figure 5A validates that higher and lower levels of nitrogen concentrations and incubation time decrease the lipid production percentage by *S. obliquus* when the initial pH is at the center value and methyl red concentrations are at lower values. Figure 5B demonstrates that the centre value of nitrogen concentrations and initial pH increased the lipid production percentage by *S. obliquus* when the incubation time was at the center value and methyl red concentrations were at low values. Figure 5C demonstrates the effect of nitrogen concentrations and methyl red concentrations on the lipid production percentage by *S. obliquus*. The results prove that the centre value of nitrogen concentrations and low value of methyl red concentrations promote the lipid production percentage by *S. obliquus* when two other variables are maintained at their centre levels. Figure 5D represents the effect of incubation time and pH on the lipid production percentage by *S. obliquus*. The results proved that when nitrogen concentrations were at center levels and methyl red at low levels, the results proved that the high and low levels of the incubation time and initial pH values decreased the lipid production percentage by *S. obliquus* when the nitrogen concentration was at its center level and methyl red concentrations were at their low levels. Figure 5E shows that the centre level of incubation time and low level of methyl red concentrations increase the lipid production percentage by *S. obliquus* at their center levels of both nitrogen concentrations and initial pH. Figure 5F denotes the effect of pH and methyl red concentrations on the lipid production percentage by *S. obliquus*. The results indicated that high and central levels of methyl red concentrations and high and low levels of pH decreased lipid production by *S. obliquus* when the nitrogen concentrations and incubation time were maintained at their center levels.

### The desirability functions

**Figure 6:**
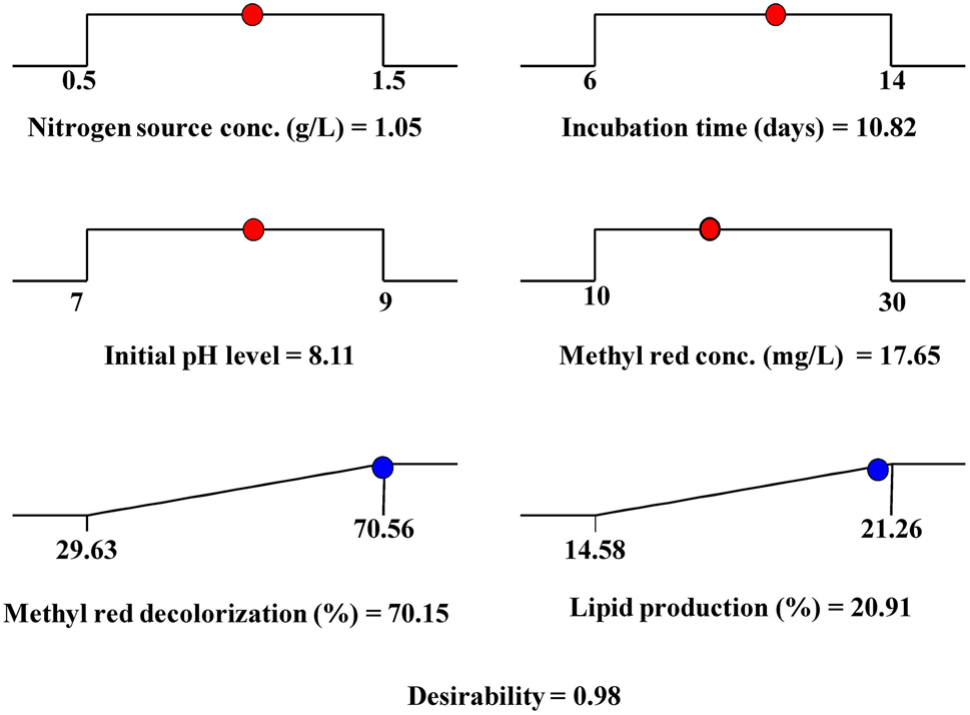
The optimization plot displays the optimum predicted values for the maximum methyl red decolorization (%) and lipid production (%) by using *Scenedesmus obliquus* and the desirability function.

One of the key targets of the experimental design analysis is to identify the optimum conditions predicted to maximize the response. Due to its simplicity, the desirability function (DF) approach has been commonly employed to determine the optimum predicted conditions for the optimization of multi-response processes^61^. The DF strategy is used to determine the optimum predicted conditions that can provide the "most desirable" response values. The use of DF will effectively contribute to the optimization of biotechnological processes with a reduced number of replicates. The function of desirability ranged between zero (indicating an undesirable response) and one (indicating a completely desirable response). For the optimization process, the DF option in Design Expert Software (version 7) was used. The optimum predicted conditions reached with a desirability function of 0.98 for the maximum decolorization of methyl red % and lipid production (70.15 and 20.91%; respectively) by *S. obliquus* (Fig. 6) were obtained by using nitrogen concentrations of 1.05 g/L medium, an incubation time of 10.82, the initial pH level of 8.11, and methyl red concentrations of 17.65 mg/L. These optimum values were confirmed experimentally, which resulted in methyl red decolorization of 70.15% and lipid production by *S. obliquus* of 20.91% dry weight.

Bisht et al.^62^ reported that one g/L sodium nitrate was added to BG11 medium to show better biomass growth and lipid productivity of *Scenedesmus* sp. DBTKU. The lipid contents of *Chlorella sorokiniana* DOE1412 were not affected by pH levels^63^. El‑Naggar et al.^64^ used Face-centered central composite design (FCCCD) to find out the optimum levels and to analyze the combined effects of initial pH, contact time, Hg^2+^, Remazol brilliant blue (RBB), and biomass concentrations on the biosorption process of Hg^2+^ and RBB dye simultaneously from the binary mixture by *Gelidium corneum* biomass. FCCCD design of experiments resulted in a maximum removal percentage of RBB of 89.18% which was obtained using 200 mg/L Hg^2+^, 100 mg/L RBB, pH 5, 4 g/L algal biomass and 180 min of contact time. As well, El‑Naggar et al.^61^ used a FCCCD based optimization to investigate the efficiency of the *Gracilaria* seaweed biomass as a sustainable biosorbent for bioremoval of methylene blue from aqueous solution. The highest bioremoval percentage of methylene blue was 94.86%, obtained under optimum experimental conditions: 6 g/L *Gracilaria* seaweed biomass, initial pH 8, 20 mg/L of methylene blue, 150 mg/L of Ni^2+^ and 180 min of contact time. On the other hand, El‑Naggar et al.^59^ used FCCCD to optimize the process variables levels and analyze their combined effects on Congo red dye removal percentage. The highest removal percentage of Congo red dye (97.89%) was achieved using 100 mg/L Congo red dye, 200 mg/L Pb^2+^, 3 g/L algal biomass, initial pH 6 and contact time was 120 min at 30 °C.

### Infrared of biomass of *S. obliquus*

**Figure 7:**
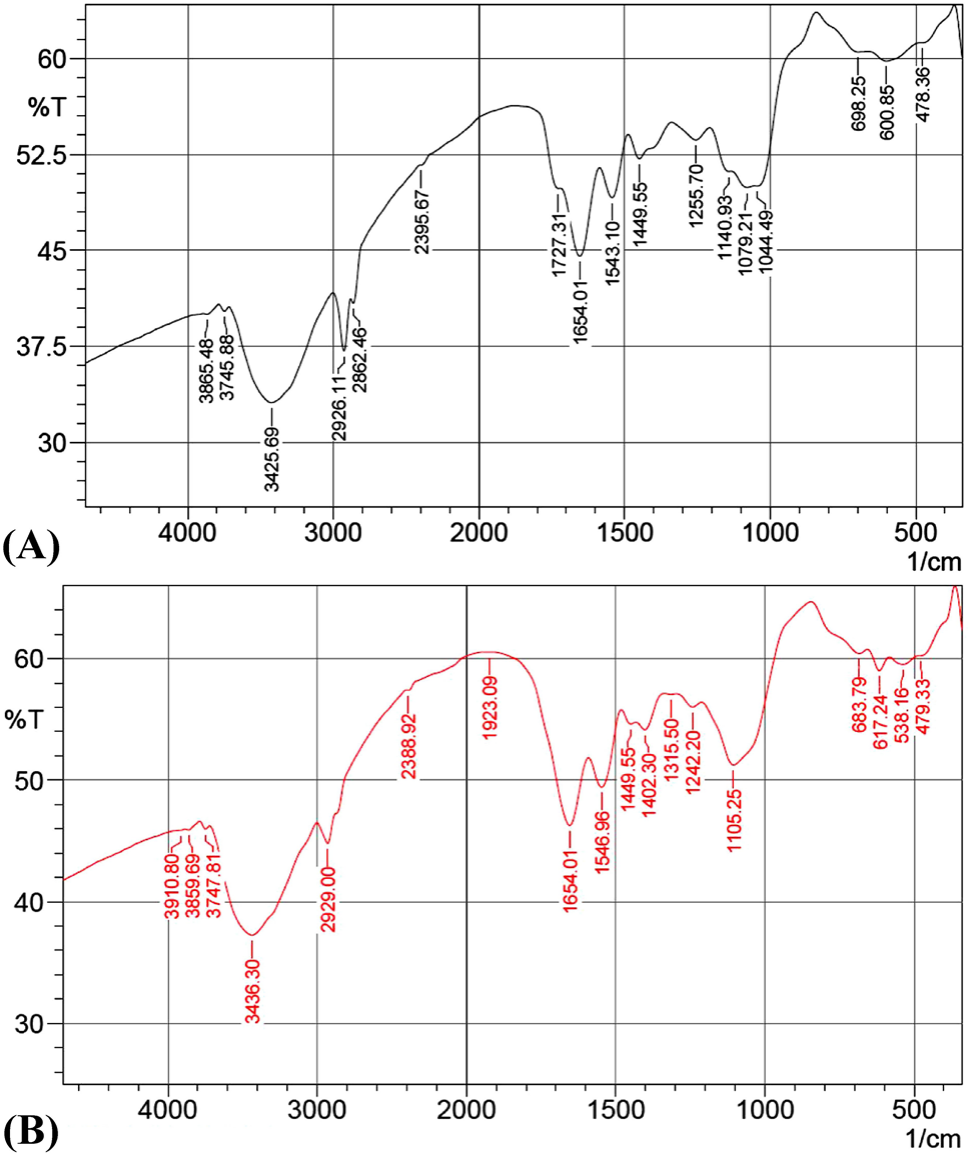
FTIR analysis of *Scenedesmus obliquus* biomass: (**A**) before and (**B**) after methyl red decolorization.

Infrared analysis of *S. obliquus* biomass was applied to identify the structural variation of the biomass after and before treatments with MR. Seventeen peaks were present in the control, and 18 peaks were present in the treated alga (Fig. 7). The peaks in control are 3865.48, 3745.88, 3425.69, 2926.11, 2862.46, 2395.67, 1727.31, 1654.01, 1543.1, 1449.55, 1255.7, 1140.93, 1079.21, 1044.49, 698.25, 600.85 and 478.36 cm^−1^, the peaks are shifted to 3910.8, 3859.69, 3747.81, 3436.3, 2929, 2388.92, 1923.09, 1654.01, 1546.96, 1449.55, 1402.3, 1315.5,1242.2, 1105.25, 683.79, 617.24, 538.16 and 479.33 cm^−1^ after treatments with MR.

Peak at 3425 cm^−1^ assigned OH stretching of alcohols and phenols^65^. The peak at 3436.3 cm^−1^ was due to the stretching vibration of OH^66^. The peaks at 2926 cm^−1^ and 2929 cm^-1^ correspond to aliphatic C–H stretching^67^. The peak at 2395 cm^−1^ represents weak absorption HPO_4_^68^. The peak at 2388 cm^−1^ denoted O–H···O–P modes (strong H-bonds)^69^. The peak at 1727 cm^−1^ was due to the stretching vibration of O–H^70^. The peak at 1923 cm^−1^ represents asymmetric stretching of alkene^71^. Peaks at 1664, 1546, 1543, 1255, and 1242 cm^−1^ correspond to amide groups^72,73^. The peak at 1449 cm^−1^ represents asymmetric CH_3_ bending of the methyl groups of proteins^74^. The peak at 1402 cm^−1^ represents CH_3_ symmetric deformation^75^, 1315 cm^−1^ amino groups^76^. Peaks at 1105 cm^−1^ represent carbohydrates^77^, and those at 1079 cm^−1^ represent phosphate groups^78^. The peak at 1044 cm^−1^ may be due to the residual 70 inorganic sulfate ions. Peaks ranged 800 to 600 represent C-Cl^79^. It is evident that there was due to the stretching vibration of the azo bond diminishes in 1654 cm^−1^, The intensity shifted after treatment by methyl red from 46.272 before treatment by methyl red to 44.541 (after treatment by methyl red), there is a new peak in the azo range (after treatment by methyl red) is 1727.31 cm^−1^, there is a little shift in the peak 1546 cm^−1^ (before treatment) to 1543 cm^−1^ (after treatment by methyl red) and the intensity peak shifted from 49.426 (before treatment) to 49.114 after treatment by methyl red.

### Scanning electron microscopy (SEM)

**Figure 8:**
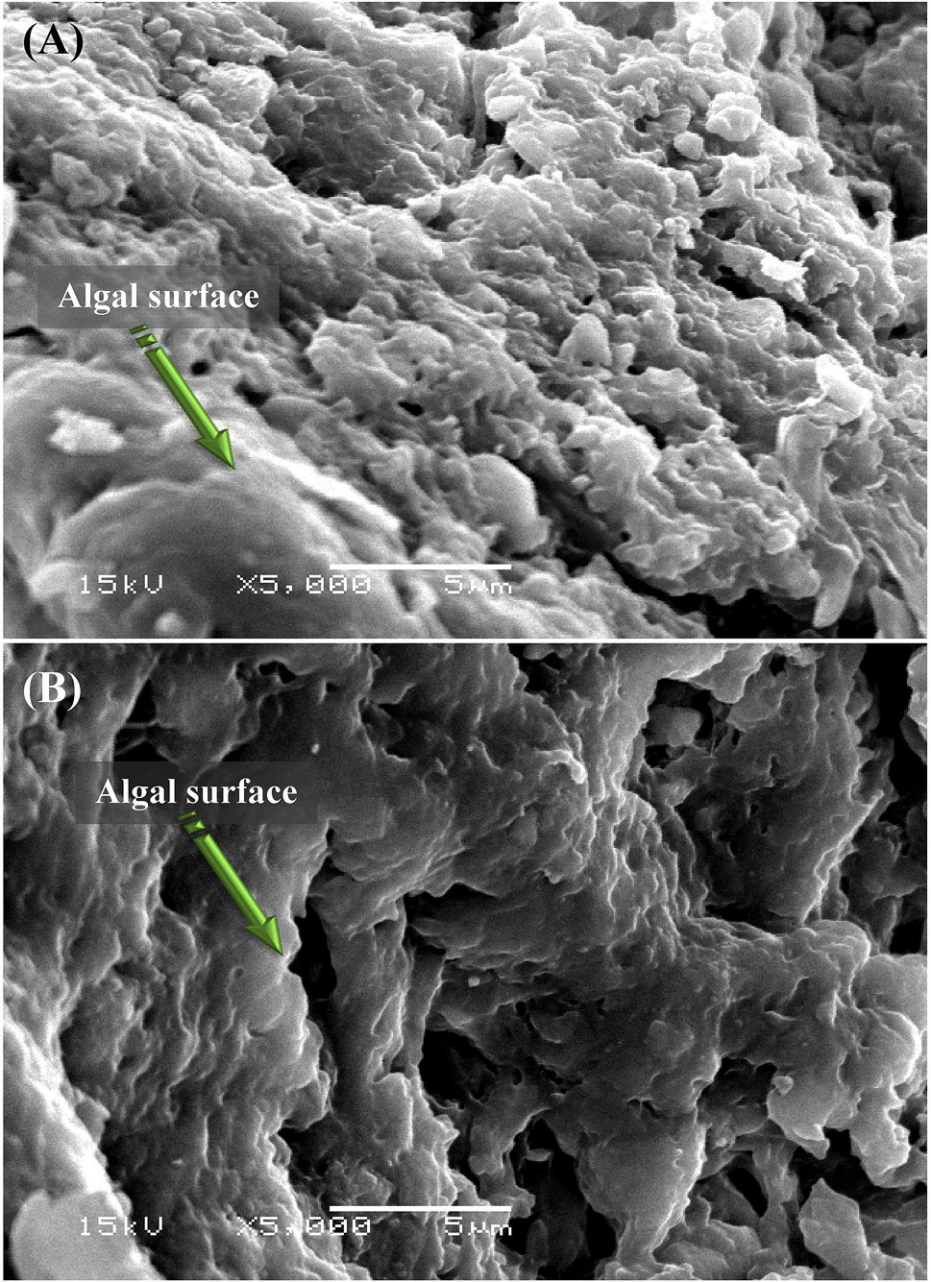
SEM micrograph of *Scenedesmus obliquus* biomass: (**A**) before and (**B**) after methyl red decolorization.

SEM micrographs of *S. obliquus* biomass after and before treatments with MR are shown in Fig. 8. The results indicated that the untreated alga was relatively smooth and no impurities were present, but in the case of alga treated with MR, there were large amounts of impurities and shrinkage. When *Chlorella s*p. absorbed lower concentrations of dyes, the cell surface became less smooth^80^.

### Lipid’s profile

**Figure 9:**
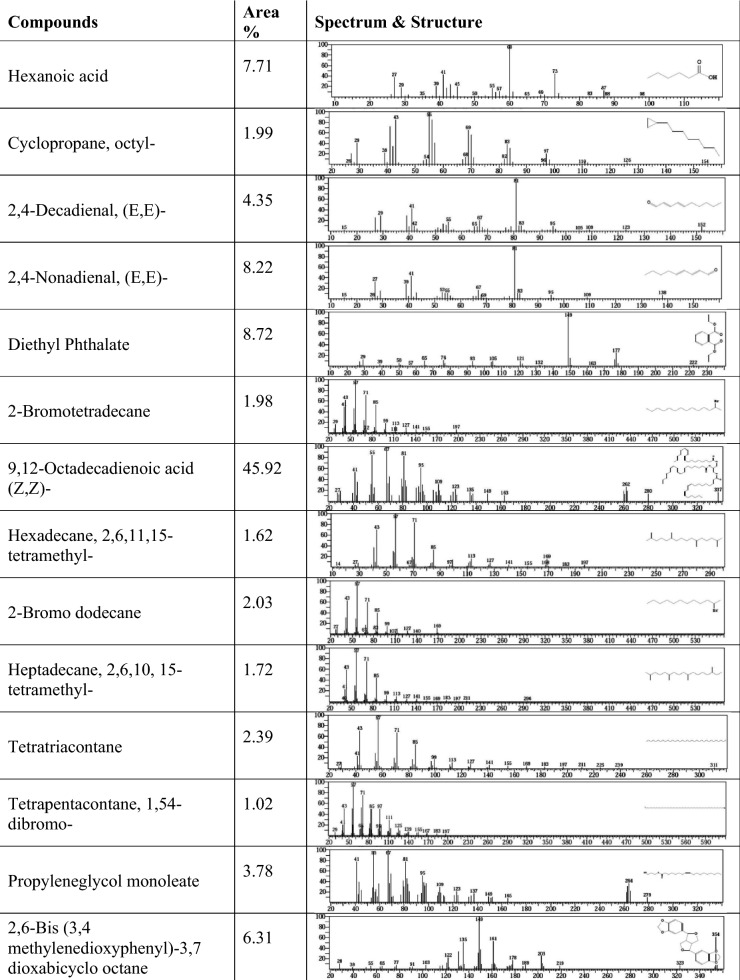
GC–MS analysis of lipids produced by *Scenedesmus obliquus* before methyl red decolorization.

**Figure 10:**
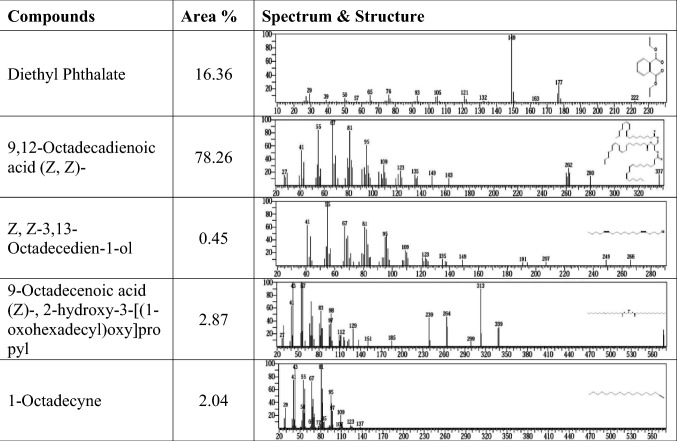
GC–MS analysis of lipids produced by *Scenedesmus obliquus* after methyl red decolorization.

Twenty compounds appeared in the lipid extract from the alga (*S. obliquus*) grown with M.R., but in the case of the control, there were only 10 compounds. This was because alga was grown with methyl red under mixotrophic conditions and used the carbon present in dye as the sole carbon source, but the control alga was grown under autotrophic conditions and CO_2_ assimilation. The results in Figs. 9 and 10 indicate that the chemical composition of the methyl esters of fatty acids extracted from *S. obliquus* was grown under mixotrophic and phototrophic conditions, respectively. Compounds are present in GC–MS profiles after methylations of hexane extracts of *S. obliquus* were grown with methyl red (mixotrophic conditions): hexanoic acid, cyclopropane, octyl-, 2,4-decadienal, (E, E)-, 2,4-nonadienal, (E, E)-, diethyl phthalate. Nonadecane, 2-bromotetradecane, 1-chloroeicosane, 9,12-octadecanoic acid (Z, Z)-, hexadecane, 2,6,11,15-tetramethyl-, hexadecane, 2-bromo dodecane, heptadecane, 2,6,10,15-tetramethyl-, tetratriacontane, hentriacicontane, tetrapententacontane, 1,54-dibromo-, (R)-(−)-14-methyl-8-hexadecyn-1-ol, propylenegal monoleate, 2,6-bis(3,4-methylenedioxyphenyl)-3,7-dioxabicyclo (3.3.0) octan, and cyclopropanebutanoic acid, 2-[[2-[[2-[(2-pentylcyclopropyl) were met. Compounds were present after hexane extract from *S. obliquus* was grown under photoautotrophic conditions were diethyl Phthalate, 9,12-octadecadienoic acid (Z, Z)-, Z, Z-3,13-octadecedien-1-ol, 9-octadecenoic acid (Z)-, 2-hydroxy-3-[(1-oxohexadecyl) oxy]pr, 1-octadecyne, 13-tetradecynoic acid, methyl ester, hexanoic acid, 2-tetradecyl ester, octanoic acid, hexadecyl ester, 2-octanol, pentafluoropropionate, and tricyclo [20.8.0.0] triacontane, 1(22),7(16)-diepoxy-. Five major fatty acids in *Scenedesmus dimorphus* were oleic acid, linolenic acid, palmitic acid and 2-methy tetracosane 16.12%, 12.68%, 10.14%, 10.11% and 6.83%, *Scenedesmus quadricauda* lipids components were palmitic acid, oleic acid, linoleic acid, 3, 7, 11- trimethyl-2,4-dodecadiene and linolenic acid (16.36%, 15.60%, 11.67%, 9.67% and 6.21%, respectively^81^. The polyunsaturated essential fatty acid 9,12-octadecadienoic acid methyl ester (Z, Z) (linoleic acid) is present in the lipid profile of *S. obliquus* when grown under autotrophic conditions and that grown under mixotrophic conditions, but the percentage of contents were differ.

The maximum amount is present in the lipids of alga grown under autotrophic conditions and represents 78.26, but in the case of alga grown in mix-trophic conditions, it represents 45.92. Therefore, the lipid composition and its percentage of alga grown with MR differed from the lipid profile of the same alga when grown under autotrophic conditions. Alga grown with methyl red had a lot of different compounds in their lipid profile than those grown under photoautotrophic conditions. This could be due to alga grown with methyl red has a different metabolism for producing fatty acids. Under mixotrophic conditions, algae use methyl red as the sole carbon source. Fazal et al.^30^ reported that microalgae utilize dyes as a carbon source and transform them into metabolites. Microalgae also work as biosorbents because dyes can be absorbed onto their surface^82^. The low amount of unsaturated fatty acids in the lipid profile of alga cultivated with methyl red may be due to the nitrogen content of the dye elevating the available nitrogen to alga. When the nitrogen deficiency reached day 10, the lipid content of *Scenedesmus* sp. increased from 14 to 31%^83^. Numerous other hydrocarbons were detected in addition to fatty acids due to the algae's metabolism being distinct from that of phototrophic algae. The microgreen algae *Chlorella pyrenoidosa* and *Chlorella vulagris* degrade dyes into simple aromatic amines and decolorize dye wastewater^84^. Therefore, it is possible to use the fatty acids extracted from alga grown with methyl red to produce biodiesel and simultaneously remove dyes.

An engine fuelled with biodiesel containing superior oxygen content can lead to lower CO emissions with increasing blend ratio due to complete combustion in the diesel engine. An engine fuelled with biodiesel containing superior cetane number and higher lubricity is more effective.

Biodiesel with higher gross calorific value produces higher power. Biodiesel has a higher viscosity, which causes fuel flow and ignition problems in engines and decreases power output^85^. Lipid yields by microalgae are controlled by various culturing conditions, such as nitrogen deprivation^86^, harvesting times^87^, and pH^88^. Additionally, the methods and solvents used to extract lipids affect lipid yields and contents^43^. The oxidation state of the nitrogen source (i.e., NH_4_ or NO_3_) can promote the influence of biomass yield^89^. Musa et al.^90^ reported that the use of NO_3_ as a nitrogen source requires high energy and diminishes biomass yield. The results indicated that unsaturated fatty acids were more abundant than saturated fatty acids. Saturated acid components in algal biomass are in the range of 25–45%, while unsaturated fatty acid contents account for 50–55% of total fatty acids^91^. Xin et al.^83^ reported the potential production of biodiesel from lipids extracted from *Scenedesmus* sp. grown in wastewater treatment. Mata et al.^92^ stated the possible production of biodiesel from *Scenedesmus obliquus* that was grown in brewery wastewater. The lipid percentage of *Scenedesmus obliquus* grown with total wastewater was 22.7, whereas the lipid percentage of algae grew in BBM 25.2^51^. Mata et al.^92^ reported that *Scenedesmus* sp. Z-4 displayed great potential to realize simultaneous wastewater treatment and lipid production at low temperatures. Table 6 reported the various factors affecting the decolorization of methyl red dye from aqueous solutions by using different sorbents (algae and bacteria). There was variation in factors (incubation time, pH, methyl red conc., temperature) influencing the decolorization of methyl red according to the biosorbents. The incubation times were 60 h, 20 m, 60 m, 24 h, 48 h and 6 h with the biosorbents *Chara vulgaris*, *Neplhelium lappaceum* seeds, *Galactomyces geotrichum* MTCC 1360, *Rhodococcus strain* UCC 0016, *Bacillus megaterium* ITBHU01 and *Spirulina*-C11; respectively at pH 5, 3, 7, 7.21 and 8 with *Chara vulgaris*, *Neplhelium lappaceum* seeds, *Rhodococcus* strain UCC 0016, *Bacillus megaterium* ITBHU01 and *Aeromonas jandaei* strain^93–100^; respectively. Many studies used algae to remove dyes and heavy metals from wastewater^29,101,102^. The lipid yields by algae can be stimulated by some factors such as ultrasonication^103^ and addition of iron oxide nanoparticles to the algae growth media^104–107^.

**Table 6:** Optimization factors for decolorization of methyl red dye from aqueous solutions by various biosorbents.

Biosorbent	Incubation time	pH	Methyl red conc., mg/L	Temp.	Decolorization %	References
*Scenedesmus obliquus*	10 days	8.11	17.65	Ambient temp.,	70. 15	This study
*Chara vulgaris*	60 h	5	40	–	–	Mahajan and Kaushal^94^
*Neplhelium lappaceum* seeds	20 m	3	690	30	–	Zein et al.^95^
*Galactomyces geotrichum* MTCC 1360	60 m	–	100	30	100	Jadhav et al.^96^
*Rhodococcus* strain UCC 0016	24 h	7		30	100	Maniyam et al.^97^
*Bacillus megaterium* ITBHU01	24	7.21	258.54	38.2	98.1	Tripathi et al.^98^
*Spirulina*-C11	48 h	–	100		65.2%	Ansari et al.^99^
*Aeromonas jandaei* strain SCS5	6 h	8	100	35 C	100%	Sharma et al.^91,100^

## Conclusions

This study demonstrates a novel approach that uses the microgreen alga *Scenedesmus obliquus* to decolorize methyl red and produce lipids for possible transesterification to biodiesel. Experimentally, the maximum methyl red removal and lipid production were 70.015 and 20.91%; respectively. The methyl red concentration was 17.95 mg/L, nitrogen concentration was 1.05 mg/L, incubation time was 10 days, and pH was 8.11. Fresh algal biomass from *S. obliquus* can be used to remove methyl red from wastewater efficiently, making lipids that can be transesterified into biodiesel and solving both fuel and environmental problems. Algae can be used in simultaneous bioremediation of wastewater and production of biofuels like biodiesel that is derived from lipid content and bio-methanol that is derived from carbohydrate content. Extensive study should be required to determine the optimal conditions for production and harvesting of high lipid content algae to make algae fuel for future energy.
